# Patients with symptomatic permanent atrial fibrillation show quantitative signs of pain sensitisation

**DOI:** 10.1136/openhrt-2021-001699

**Published:** 2021-06-17

**Authors:** Adam Jackson, Ole Frobert, Dennis Boye Larsen, Lars Arendt-Nielsen, Anna Björkenheim

**Affiliations:** 1 Department of Cardiology, Örebro University Hospital, Sweden, Örebro, Sweden; 2 Department of Cardiology, Faculty of Medicine and Health, Örebro University, Sweden, Örebro, Sweden; 3 Department of Health Science and Technology and the Center for Sensory-Motor Interaction/Center for Neuroplasticity and Pain, Faculty of Medicine, Aalborg University, Aalborg, Denmark

**Keywords:** atrial fibrillation, chest pain, obesity

## Abstract

**Objective:**

Most patients with atrial fibrillation (AF) report symptoms, while one-third are asymptomatic. We hypothesised that sensory processing, in particular pain, differs in patients with symptomatic and asymptomatic AF.

**Methods:**

Thirty individuals with permanent AF (15 symptomatic and 15 asymptomatic) completed the Atrial Fibrillation 6 (AF6) and short form 36 Health Survey questionnaires and underwent quantitative pain sensitisation testing using pressure algometry at the sternum (referred pain area) and the tibialis anterior muscle (generalised pain area). The primary objective was to assess differences in pressure pain thresholds (PPT), temporal summation of pain (TSP) and conditioned pain modulation (CPM) in the two groups. The secondary objective was to determine association of demographic and clinical parameters to measures of pain sensitisation.

**Results:**

The symptomatic group had lower PPTs at both tibialis (p=0.004) and sternum (p=0.01), and impaired CPM (p=0.025) and facilitated TSP (p=0.008) at the tibialis but not sternum, compared with the asymptomatic group. The AF6 sum score was negatively correlated to PPT on both tibialis (r=−0.50, p=0.005) and sternum (r=−0.42, p=0.02) and positively correlated to TSP on both tibialis (r=0.57, p=0.001) and sternum (r=0.45, p=0.01), but not to CPM. The physical component summary score was positively correlated to the PPT on both tibialis (r=0.52, p=0.003) and sternum (r=0.40, p=0.03) and negatively to TSP on the tibialis (r=−0.53, p=0.003) but not sternum.

**Conclusions:**

Patients with symptomatic AF exhibit lower pain tolerance than patients with asymptomatic AF, as well as impaired pain inhibitory control and facilitated summation of pain, indicating that pain sensitisation may be of importance in symptomatic AF.

Trial registration number

NCT04649437.

**Key questions**

**What is already known about this subject?**

- It is unclear why some individuals with atrial fibrillation (AF) experience symptoms whereas others are asymptomatic, and little is known of the physiological mechanisms underlying AF symptomology. Previous studies have found that symptomatic AF is more frequent in women, at younger ages, and in patients with non-permanent AF. It is possible that patients with symptomatic AF differ in pain processing from those not perceiving symptoms.

**What does this study add?**

- Patients with symptomatic AF show significant signs of pain sensitisation compared with patients with no perceived symptoms of AF.

**How might this impact on clinical practice?**

- Our findings suggest there is potential for investigation of therapies to target pain sensitisation in patients with symptomatic AF.

## Introduction

Approximately one-third of individuals with atrial fibrillation (AF) report no symptoms, but up to 25% report severe symptoms such as palpitations, dyspnoea, chest pain and syncope.^1 2^ In studies of patients with permanent AF, 9%–15% reported chest pain.^3 4^ About one-third of patients with symptomatic AF also suffer from psychological distress.^5 6^ It remains unclear why some individuals with AF are asymptomatic, whereas others are severely symptomatic. Patients with chronic pain conditions such as osteoarthritis, chronic pancreatitis and irritable bowel syndrome show a high degree of pain sensitisation, that is, facilitated pain responses to repeat painful stimulation and impaired pain inhibitory control compared with controls.^7–9^ It is not known whether patients with symptomatic AF exhibit a high degree of pain sensitisation in the absence of chest pain as a symptom. We hypothesised that pain sensitisation is an important factor driving symptom perception in AF. The primary objective of this study was to assess differences in pain sensitisation in patients with symptomatic and asymptomatic AF. The secondary objective was to analyse associations of demographic (age, sex and body mass index (BMI)) and clinical parameters (AF duration, Atrial Fibrillation 6 (AF6) sum score and physical and mental component summary scores of the short form 36 Health Survey (SF-36) with the quantitative measures of pain sensitisation.

## Methods

### Study population

Study participants were recruited from a database consisting of all individuals aged 20 years or older with an initial or recurrent AF diagnosis between 1 January 2015 and 31 December 2018 in Örebro County, Sweden. The database comprised patients derived from the Medrave 4 Registry for primary care, the Auricula Registry (national quality registry for anticoagulation treatment) and the National Patient Registry for inpatient and specialised outpatient care. Eligibility criteria for the current study were ≤75 years old, permanent AF (persistent AF for which no further attempts to restore sinus rhythm will be undertaken^10^) and a previously completed AF6 questionnaire^11^ in spring of 2021. Reasons for exclusion were pregnancy, diabetes mellitus, previous AF ablation, psychiatric condition or cognitive impairment, drug or alcohol abuse, previous or concomitant neurological musculoskeletal disorder or continuous analgesic medication. Candidate participants received written study information and were offered an outpatient visit to a physician (AB) at the Department of Cardiology, Örebro University Hospital. Patients were asked not to take analgesic medication within the 24 hours preceding the visit. After obtaining written informed consent, patients were asked to repeat the AF6 and SF-36^12^ questionnaires, rate their chest pain symptoms on the Visual Analogue Scale (VAS) and draw the area of pain on a body chart. Comorbidities, medications, duration of AF and ranking on the modified European Heart Rhythm Association (EHRA) symptom scale^13^ were documented, and a routine clinical examination was performed including a 12-lead ECG. All data were obtained during the visit and no follow-up visits were conducted. Subjects were divided into two groups based on their earlier AF6 sum scores: 15 asymptomatic patients with an AF6 sum score of 0, and 15 symptomatic patients with an AF6 sum score of ≥30. The cut-off of 30 was based on a study showing patients scheduled for AF ablation, who are generally highly symptomatic, to have a median score of 30 points.^14^ Sample size flat rules of thumb was used as this was a proof-of-concept study.^15^ The study was conducted in accordance with the Declaration of Helsinki.

### Symptom assessment and health-related quality of life

The patient-reported AF6 questionnaire has a recall period of 7 days and includes breathing difficulties at rest, breathing difficulties on exertion, limitations in day-to-day life, feeling of discomfort, tiredness and worry and/or anxiety due to AF. A score of 0 (no symptoms) to 10 (severe symptoms) is reported for each of the six items and scores are added to obtain a cumulative sum score ranging from 0 to 60, with higher values reflecting more severe symptoms.^11 16^

The physician-assessed modified EHRA scale was used to assess symptom severity relating specifically to the time when patients feel symptoms of AF. Patients reporting no AF symptoms are categorised as class I. Class IIa experienced mild, class IIb moderate, class III severe and class IV, disabling symptoms.^13^

The patient-reported SF-36 has a recall period of 4 weeks and consists of 36 items assessing eight domains on a scale ranging from 0 to 100, with higher values indicating better health-related quality of life. The eight domains generate a physical component summary score and a mental component summary score, which were used to assess health-related quality of life.^12^

### Quantitative sensory testing

Quantitative sensory tests were conducted by a blinded investigator (AJ) in the following order: pressure pain threshold (PPT), temporal summation of pain (TSP) and conditioned pain modulation (CPM). PPTs and TSP were performed at the tibialis anterior muscle 5 cm distal to the tibial tuberosity, and at the sternum between the third and fifth intercostal spaces, with 1 min intervals between repeats and sites.^17^ The sites were chosen to reflect localised (sternum, referred pain area) and generalised (tibialis anterior muscle) manifestations of sensitisation. Patients were free to stop the test at any time.

### PPT

The PPT was defined as the minimum pressure required for the sensation of pressure to change to pain. The 1 cm^2^ probe of the handheld pressure algometer (Somedic AB, Sweden) was placed perpendicular to the skin, and pressure was increased at a rate of approximately 30 kPa/s. Participants were instructed to press the stop button on the algometer when they first experienced the sensation of pain. The mean of three measurements at each site was used for analysis.

### TSP

A pin-prick device (0.25 mm^2^ tip) with a weighted load (Aalborg University, Denmark) was used to induce TSP.^18 19^ A force of 60 g was applied to both sites, and patients were asked to rate the pain intensity on a VAS (0 to 10). Ten consecutive stimuli were applied with a 1 s interval between stimuli and the subjects were asked to rate the pain intensity of the final stimulation on the VAS. Temporal summation of pain was defined as the difference in pain intensity between the first and the final stimulation. Higher TSP scores indicated facilitated temporal summation.

### CPM

The right hand was immersed up to the wrist in circulating water at 2°C–4 °C for up to 2 min (cold pressor test). The PPT from both sites were assessed before (unconditioned) and after (conditioned) the 2 min immersion and reassessed 10 minutes after the hand was withdrawn from the water. The CPM was calculated as the absolute difference in PPT during the immersion compared with the pre-immersion PPT. A negative value indicates inefficient CPM and a positive value indicates efficient CPM.

### Statistical analysis

Data were tested for normality using the Shapiro-Wilk’s test. Independent sample t-tests were performed to analyse differences between groups in age, BMI, AF duration and physical/mental component summary scores, and dependent sample t-tests to quantify difference in initial and repeat AF6 sum scores.

The χ^2^ test was employed to test for differences in sex, comorbidities, rate control agents (beta-blockers, verapamil, diltiazem and digoxin individually or combined), and completion of the full 2 min immersion cold pressor test between the two groups. A mixed model analysis of variance (ANOVA) was used to assess the primary objective of the study, with within-factor time (baseline, 2 min and 10 min), and between groups. One-way ANOVA was used to characterise differences in CPM and TSP. To adjust for age, sex and BMI, these variables were included as covariates for CPM and TSP. Sidak-corrected post hoc tests were employed where appropriate, if main or interaction factors were significant. Secondary objectives were tested using correlational analyses, Pearson’s product-moment or Spearman’s rank correlation where appropriate for demographic and clinical parameters, and pain sensitisation as reflected by PPT, CPM and TSP. A p<0.05 was considered significant. Data were reported as mean±SEM unless otherwise stated. Statistical analyses were performed using IBM SPSS Statistics V.27.

### Patient and public involvement

Patients and public were not invited to comment on the study design and were not consulted to develop outcomes relevant to patients or to interpret the results. Patients were not invited to contribute to the writing or editing of this document for readability or accuracy. Before publication, the results of this study were not disseminated to patients or the public.

## Results

### Study population—clinical characteristics

Thirty patients were enrolled in the study between November 2020 and January 2021 (mean age 69±6 years, 60% men, duration of AF 7±4 years) (table 1).

**Table 1:** Characteristics of patients with symptomatic versus asymptomatic permanent AF

	Symptomatic (n=15)	Asymptomatic (n=15)
Male—n (%)	8 (53)	10 (67)
Age—years, mean (SD)	69 (7)	69 (4)
Body mass index—kg/m^2^	32 (7)	25 (3)
Concomitant disease—n (%)		
Hypertension	10 (67)	10 (67)
Heart failure	0	0
Coronary artery disease	1 (7)	0
Peripheral artery disease	0	0
History of stroke or TIA	0	2 (13)
Chronic kidney disease	0	0
Obstructive sleep apnoea	4 (27)	4 (27)
AF history and symptoms		
Duration of AF—years, mean (SD)	7 (4)	7 (4)
Heart rate at rest—beats/min, mean (SD)	80 (13)	71 (12)
CHA_2_DS_2_-VASc scores, mean (SD)	1.9 (1.3)	1.7 (0.9)
AF6 sum score inclusion, mean (SD)	30 (11)	0
EHRA symptom scale class n (%)		
I	0 (0)	15 (100)
IIa	8 (53)	0
IIb	5 (33)	0
III	2 (13)	0
IV	0	0
Medications—n (%)		
Oral anticoagulation	13 (87)	14 (93)
Beta-blockers	14 (93)	10 (67)
Verapamil or diltiazem	1 (7)	0
Digoxin	1 (7)	0
ARB or ACE inhibitor	10 (67)	8 (53)

AF6, Atrial Fibrillation 6; AF, atrial fibrillation; ARB, angiotensin receptor blocker; CHA_2_DS_2_-VASc, congestive heart failure, hypertension, age ≥75 years (doubled), diabetes, prior stroke or transient ischaemic attack (doubled), vascular disease, age 65–74, female; EHRA, European Heart Rhythm Association; TIA, transient ischaemic attack.

The groups were well matched, except for a significantly higher BMI in the symptomatic group (t (17.8)=−3.73, p=0.002). The resting heart rate was adequately rate controlled in all patients with no significant differences between the groups and no significant differences in treatment with rate control agents.

### Symptoms of AF and health-related quality of life

Patients in the symptomatic group had significantly lower AF6 sum scores at inclusion in the study (30±11) than recorded in spring of 2020 (38±8, t=2.97, p=0.01), while all asymptomatic patients had an AF6 sum score of 0 both times. The item consistently scoring as most severe was breathing difficulties on exertion (table 2).

**Table 2:** Atrial fibrillation symptoms in symptomatic patients with permanent atrial fibrillation using the AF6 questionnaire

AF6 item	Patients contributing to the score n	Score, mean (SD)
Breathing difficulties at rest	13/15	3.2 (2.6)
Breathing difficulties on exertion	15/15	7 (2.6)
Limitations in day-to-day life	15/15	5.3 (2.8)
Feeling of discomfort	12/15	4.2 (3.4)
Tiredness	15/15	5.9 (3)
Worry and/or anxiety	13/15	4.4 (3)

AF6, Atrial Fibrillation 6.

Patients with symptomatic AF were considered to be in EHRA class IIa–III, whereas all asymptomatic patients were considered to be in EHRA class I. Symptomatic patients reported significantly lower physical and mental component summary scores compared with asymptomatic patients (t(19.19)=8, p<0.001 and t(21.696)=5.27, respectively, p<0.001).

**Figure 1:**
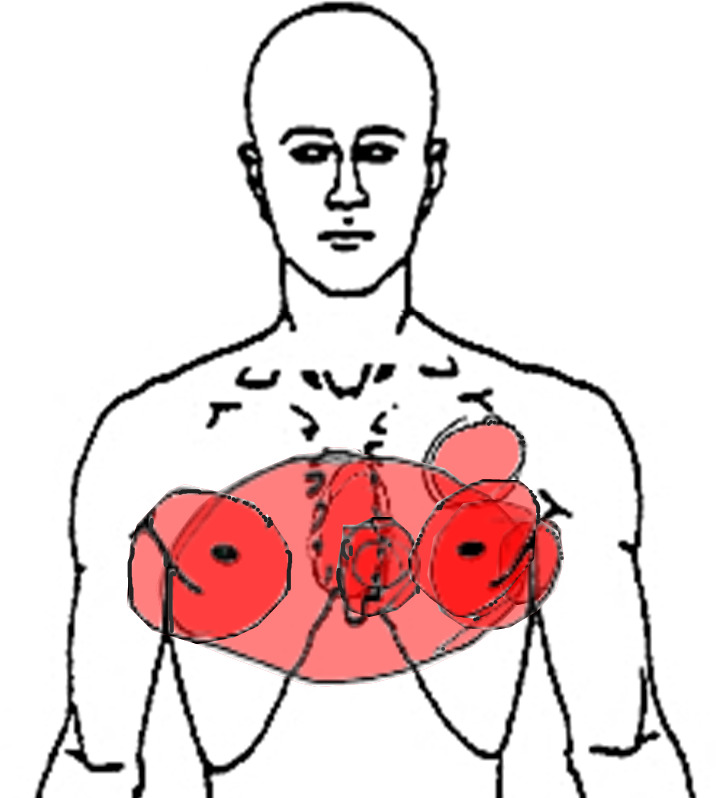
Superimposed body chart (n=4) of chest pain distribution in patients with permanent atrial fibrillation.

Four patients (13%) reported chest pain at some point during the prior 7 days, all of whom were in the symptomatic group (VAS 4, 6, 8 and 8). A body chart with superimposed chest pain distribution is shown in figure 1. Three of the patients who reported chest pain had previously undergone coronary angiography or radionuclide myocardial perfusion imaging with normal results. The fourth patient had undergone percutaneous coronary intervention due to myocardial infarction 5 years before diagnosis of AF.

### Quantitative sensory testing

An overview of quantitative sensory testing values is available as online supplemental material.

10.1136/openhrt-2021-001699.supp1 Supplementary data

### Pressure pain threshold

**Figure 2:**
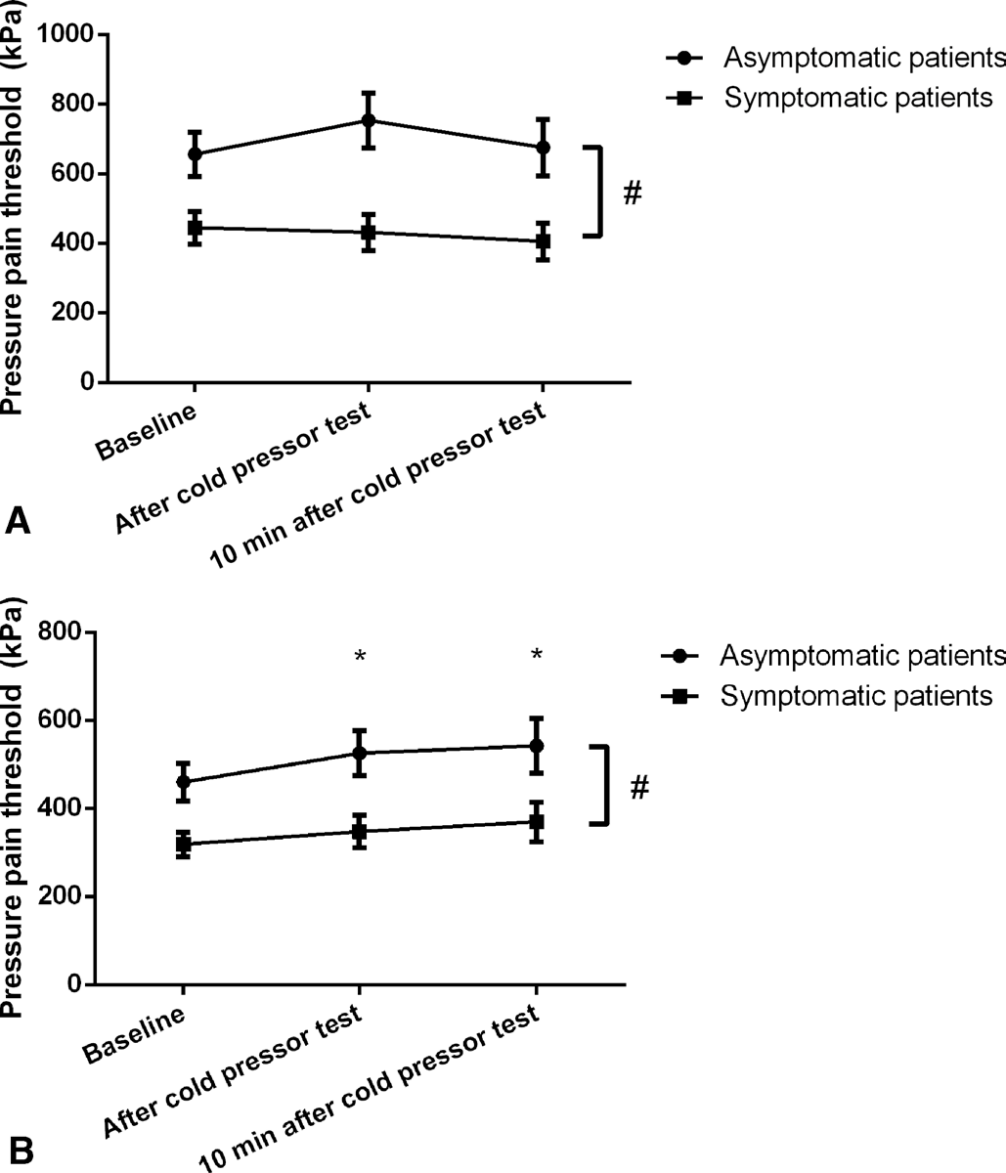
Pressure pain thresholds (PPTs) before and after the cold pressor test. PPTs over the tibialis anterior muscle (A) and the sternum (B) before and immediately after cold pressor test in patients with symptomatic and asymptomatic permanent atrial fibrillation (AF). Values are mean and SEM of three measurements. #, sternum and tibial PPTs differed significantly in asymptomatic and patients with symptomatic AF, p<0.05. *, sternum PPTs were significantly increased immediately after and 10 min after cold pressor test compared with baseline, p<0.05.

The mixed model ANOVA did not reveal significant interaction between time and group (*F*_1.34,37.53_ =0.52, p=0.53, η^2^=0.02) but a significant main effect of time (*F*_1.34,37.53_ =6.11, p=0.01, η^2^=0.18) on sternum PPTs was found. Post hoc between-group analysis showed significantly lower PPTs on the sternum for symptomatic patients (*F*_1,28_ =7.49, p=0.01, η^2^=0.21), and PPTs were significantly increased 2 min (p=0.02) and 10 min (p=0.04) after hand immersion overall (figure 2). Similarly, no significant interaction was found for time and group for PPTs on the tibialis anterior muscle (*F*_1.57,44.05_ =2.13, p=0.14, η^2^=0.071) or main effect of time (*F*_1.57,44.05_=2.11, p=0.14, η^2^=0.07). Patients with symptomatic AF reported lower PPTs over the tibialis anterior muscle than did the asymptomatic group (p=0.004).

### Temporal summation of pain

When adjusting for age, sex and BMI, we found higher TSP in data of the tibialis anterior muscle in the symptomatic group when compared with the asymptomatic group (p=0.008). There was no significant difference between asymptomatic and symptomatic AF patients in sternum TSP (p=0.13).

### Conditioned pain modulation

All asymptomatic patients kept their hand immersed in the cold water for the full 2 min, while those in the symptomatic group withdrew their hand significantly sooner (χ^2^=6, p=0.01). Sternum CPM, assessed by one-way ANOVA adjusted for age, sex and BMI, was not significantly different between groups (p=0.16). Conversely, CPM over the tibialis anterior muscle, adjusted for age, sex and BMI, was significantly higher in the asymptomatic group compared with the symptomatic group (p=0.025). No patient showed angina pectoris, bradycardia, or syncope during the cold pressor test.

### Correlation analyses

We found a significant negative correlation between the AF6 sum score and PPTs of both the tibialis anterior muscle (r=−0.50, p=0.005) and the sternum (r=−0.42, p=0.02). There was a significant positive correlation of the AF6 sum score with TSP of both the tibialis anterior muscle (r=0.57, p=0.001) and the sternum (r=0.45, p=0.01). There were significant correlations between the physical component summary scores and the PPTs of both the tibialis anterior muscle (r=0.52, p=0.003) and the sternum (r=0.40, p=0.03) and significant negative correlation with TSP of the tibialis anterior muscle (r=0.53, p=0.003) but not the sternum. We observed significant positive correlation between the mental summary scores and the CPM of the tibialis anterior muscle (r=0.40, p=0.03) but not of the sternum or other measures of pain sensitisation. Male sex was significantly positively correlated to PPT of the tibialis anterior muscle (r=0.42, p=0.02) and CPM of the sternum (r=0.49, p=0.006).

## Discussion

The study showed significant quantitative signs of local and general pain sensitisation in patients with permanent symptomatic AF as compared with patients with asymptomatic AF, and significant correlation between quantitative pain sensitisation parameters and AF symptom severity scores.

### Symptoms of AF

Little is known about the pathophysiological mechanisms underlying AF symptomatology.^20^ Several studies have evaluated the relationship between patient characteristics and the presence of AF symptoms, and have found that asymptomatic AF is more frequent in men than in women, in older age and in patients with permanent AF.^20–22^ Furthermore, episodes of asymptomatic AF are common even in highly symptomatic patients, and AF interventions such as AF ablation increase the proportion of asymptomatic to symptomatic AF episodes.^23^ Symptoms of AF are highly variable in individual patients at different points in time,^20^ which is confirmed in the present study by the significant change in AF6 sum scores for patients with symptomatic AF from roughly 6 months before the study to inclusion. We chose 30 points as the AF6 sum score cut-off for symptomatic patients, and this corresponded well with a median score of 28 at inclusion.

This study included patients with permanent AF to avoid taking into account if patients had sinus rhythm or AF during quantitative sensory testing in this first proof-of-concept study, even though patients with permanent AF are the least symptomatic compared with patients with paroxysmal and persistent AF. It is possible that even greater differences in pain sensitisation would have been observed in a study including patients with non-permanent AF. An AF duration of 7 years is long, and patients tend to adapt to symptoms.^20^ We cannot rule out that patients in the asymptomatic group initially experienced symptoms. However, all asymptomatic patients reported an AF6 sum score of 0 both 6 months prior to the study and at inclusion.

The groups were well matched, although the symptomatic group had a higher BMI. Obesity is a known risk factor for AF and obese patients have more diastolic dysfunction, sympathicotonia and increased fatty and inflammatory infiltration of the atria compared with normal-weight patients with AF.^24^ In addition, due to low-grade inflammation, obese patients show signs of generalised pain sensitisation,^25^ which may to some degree contribute to pain processing in the patients with symptomatic AF with higher BMI. Considerable weight reduction has been shown to reduce both AF recurrences and symptoms in patients with non-permanent AF.^26^

### Quantitative sensory testing

The present study showed lower pressure pain thresholds over the referred pain area to the heart (sternum) and a distant area (tibialis anterior muscle) indicating both local and widespread pain sensitisation in patients with symptomatic AF.

The facilitated TSP in humans most likely reflects increased gain of the dorsal horn neurons, as shown in other animals.^27^ The CPM most likely reflects the balance between the descending pain inhibitory and faciliatory pathways.^19 28 29^ Both TSP and CPM reflect central changes in pain processing as involved in many chronic pain conditions.^28^ The mechanisms of pain sensitisation in humans are not fully understood, but for patients with symptomatic AF there may be links to other visceral disorders where pain is facilitated.^9 30^

The AF6 sum score demonstrated strong correlations with both TSP (positive) and PPT (negative), but no significant correlations to CPM. This could indicate an association between AF symptom severity and pain amplification but warrants further study.

The study raises important questions: (1) does AF affect pain mechanisms and increase pain sensitivity per se, or is pain sensitivity an individual trait; (2) are some patients with AF a priori more pain susceptible than others; and (3) does pain sensitisation persist during periods without AF in patients with non-permanent AF?

### Clinical relevance and limitations

In addition to existing treatment options for symptom relief such as rate control and lifestyle modifications, this study suggests evaluation therapies targeting pain and pain sensitisation in patients with symptomatic AF. Our methods should be replicated in larger cohorts and in patients with non-permanent AF.

This study has several limitations. It included a small sample size, but differences were identified between symptomatic and asymptomatic groups. Second, and possibly more important, we lacked a control group without AF and the study should be considered hypothesis generating. Finally, we excluded patients with neurological and musculoskeletal disorders and diabetes to minimise the risk that some patients could be sensitised due to other conditions, but we cannot exclude the possibility that patients had unstated previous or intermittent pain conditions.

## Conclusions

Patients with symptomatic AF show substantial signs of quantitative pain sensitisation compared with patients with asymptomatic AF, and pain sensitisation increases with an AF symptom severity score. This suggests the need for new management strategies to modulate symptoms of AF.

## Data Availability

Data are available upon reasonable request.
