# Supplementary material for: Patients with symptomatic permanent atrial fibrillation show quantitative signs of pain sensitisation
Source: Open Heart. 2021 Jun 17;8(1):e001699. doi: 10.1136/openhrt-2021-001699 (PMC8212408; doi:10.1136/openhrt-2021-001699)
Supplement: Supplementary data [file openhrt-2021-001699supp001.pdf]

Supplemental Table 1. Result of quantitative sensory testing on the tibialis anterior muscle and sternum in symptomatic and asymptomatic patients with permanent atrial fibrillation

	Symptomatic (n=15) Mean (SD)	Asymptomatic (n=15) Mean (SD)
**Pressure pain thresholds** – kPa		
Tibialis anterior muscle baseline	445 (181)	656 (244)
Tibialis anterior muscle after CPT	431 (199)	753 (307)
Tibialis anterior muscle 10 min after CPT	405 (202)	676 (312)
Sternum baseline	319 (104)	460 (169)
Sternum during CPT	348 (141)	526 (198)
Sternum 10 min after CPT	369 (171)	543 (240)
**Temporal summation of pressure pain** – VAS		
Tibialis anterior muscle	3.1 (1.6)	1.5 (1.6)
Sternum	4.3 (2.2)	3.1 (2.1)
**Conditioning pain modulation**		
Tibialis anterior muscle	-13 (137)	96 (156)
Sternum	29 (81)	66 (98)

CPT = cold pressor test; VAS = visual analogue scale.

Jackson A, *et al. Open Heart* 2022; 8:e001699. doi: 10.1136/openhrt-2021-001699

BMJ Publishing Group Limited (BMJ) disclaims all liability and responsibility arising from any reliance placed on this supplemental material which has been supplied by the author(s)

Supplemental Table 2. Relationship of baseline variables with pain sensitisation in symptomatic and asymptomatic patients (n=30) with permanent atrial fibrillation

	Correlation coefficient	P
**PPT baseline tibialis anterior muscle**		
Age	-0.18	0.33
Male	0.42	**0.02**
BMI	-0.12	0.5
AF duration	-0.33	0.08
AF6 sum score	-0.50	**0.005**
Physical component summary scores	0.52	**0.003**
Mental component summary scores	0.17	0.4
**PPT baseline sternum**		
Age	-0.10	0.6
Male	0.28	0.1
BMI	-0.17	0.4
AF duration	-0.24	0.2
AF6 sum score	-0.42	**0.02**
Physical component summary scores	0.40	**0.03**
Mental component summary scores	0.33	0.08
**TSP tibialis anterior muscle**		

Jackson A, *et al. Open Heart* 2022; 8:e001699. doi: 10.1136/openhrt-2021-001699

	23-Mar	BMJ Heart
Age	-0.14	0.5
Male sex	0.14	0.4
BMI	0.18	0.35
AF duration	-0.27	0.2
AF6 sum score	0.57	**0.001**
Physical component summary scores	-0.53	**0.003**
Mental component summary scores	-0.42	**0.02**
**TSP sternum**		
Age	-0.16	0.4
Male	0.16	0.4
BMI	0.15	0.4
AF duration	-0.36	0.05
AF6 sum score	0.45	**0.01**
Physical component summary scores	-0.26	0.2
Mental component summary scores	-0.32	0.08
**CPM tibialis anterior muscle**		
Age	0.16	0.4
Male	0.29	0.1
BMI	-0.075	0.7
AF duration	-0.045	0.8
AF6 sum score	-0.36	0.05

Physical component summary scores	0.25	0.2
Mental component summary scores	0.40	**0.03**
**CPM sternum**		
Age	0.21	0.3
Male	0.49	**0.006**
BMI	0.054	0.8
AF duration	-0.28	0.1
AF6 sum score	-0.25	0.2
Physical component summary scores	0.33	0.2
Mental component summary scores	0.14	0.5

Correlational analyses using Pearson’s product-moment or Spearman’s rank correlation where appropriate. AF6 = Atrial fibrillation 6 questionnaire; CPM = Conditioned pain modulation; PPT = Pressure pain threshold; TSP = Temporal summation of pressure pain.
